# A Mixed-Methods Study to Evaluate Elementary School Staff’s Acceptability, Delivery Challenges, and Communication Regarding the Implementation of School-Located Influenza Vaccination Program in Hong Kong

**DOI:** 10.3390/vaccines9101175

**Published:** 2021-10-14

**Authors:** Qiuyan Liao, Meihong Dong, Jiehu Yuan, Wendy Wing Tak Lam, Benjamin J. Cowling, Hau Chi So, Dennis Kai Ming Ip

**Affiliations:** 1 Division of Behavioural Sciences, Li Ka Shing Faculty of Medicine, School of Public Health, The University of Hong Kong, Hong Kong, China; meihong@hku.hk (M.D.); jhyuan@connect.hku.hk (J.Y.); wwtlam@hku.hk (W.W.T.L.); 2 Center for Infectious Disease Epidemiology and Control Collaborating with World Health Organization, Li Ka Shing Faculty of Medicine, School of Public Health, The University of Hong Kong, Hong Kong, China; bcowling@hku.hk (B.J.C.); haso9150@hku.hk (H.C.S.); dkmip@hku.hk (D.K.M.I.); 3 Laboratory of Data Discovery for Health, Hong Kong Science and Technology Park, Hong Kong, China

**Keywords:** school-located influenza vaccination, acceptability, delivery, communication

## Abstract

This was a mixed-methods study comprising a questionnaire-based survey, a qualitative study, and analysis of school newsletters to evaluate elementary school staff’s acceptability, delivery challenges and communication about school-located influenza vaccination program (SIVP) in Hong Kong. We found that school staff with lower intention to implement SIVP perceived greater logistical difficulties in arranging SIVP. Challenges regarding program delivery included schools’ limited infrastructure, the burden of paperwork, the fear of being overwhelmed by multiple school-based vaccination schedules, lacking confidence in communicating with parents about influenza vaccines, and the difficulties in managing vaccination-related anxiety among children with intellectual disability. School staff were generally passive in communicating with parents and students about influenza vaccines. We also found that schools may use the school newsletters as a substitute of the formal informed consent forms. Good partnerships among government, service providers and schools should be established to minimize the burden of paperwork for school staff, facilitate early planning of SIVP, and support schools with limited infrastructure and the vaccination of children with intellectual disabilities. Training is needed to enhance school staff’s confidence in communicating with parents and students about influenza vaccines and improve information delivery to support parents’ informed decisions for children’s vaccination.

## 1. Introduction

Seasonal influenza easily attacks children, with an annual attack rate of ~20% among unvaccinated children [1], and was associated with 16.4% of the hospitalizations among children aged 5–17 years [2]. Vaccinating school-aged children against seasonal influenza can significantly reduce the burden of influenza-like illness in children [3,4] and confer protection to a broader community [5,6]. School-located influenza vaccination program (SIVP) was evidenced to be efficient for promoting vaccination uptake rates among school-aged children elsewhere [3,4,6,7] and acceptable for elementary schools and parents in the context of the United States [8,9,10]. However, the acceptability, delivery challenges, and communication about SIVP in the Asian context remained underexplored, which raised questions about the sustainability of the program in relevant contexts [11].

### 1.1. The Hong Kong Context

In Hong Kong, seasonal influenza was associated with 24.7% of pediatric hospitalizations [12], which were estimated to have caused 662–1046 and 214–336 days of school absenteeism and parental work loss, respectively, per 10,000 population per year [13]. As a strategy to promote seasonal influenza vaccination (SIV) uptake among school-aged children, the Hong Kong government initiated a pilot school-located influenza vaccination program (SIVP) in 2018 to provide free SIV for elementary school students, which was expanded to cover all Hong Kong elementary schools in 2019–2020 [14]. With the implementation of SIVP in Hong Kong, the uptake rates of SIV among children aged 6–12 years increased from ~30% in 2016–2017 to 55.4% in 2018–2019 and 68.1% in 2019–2020 [15]. The contexts in which SIVP was implemented in Hong Kong may differ from those in the United States in several aspects. First, two types of vaccine candidates were currently available for SIVP, the inactivated influenza vaccine (IIV) administered via injection and the live attenuated influenza vaccine (LAIV) administered via nasal spray. A meta-analysis indicates that the overall efficacy of LAIV was 44.3% (95% CI: 39.5–54.4%), but some studies reported efficacy of more than 75% for preventing hospitalization in healthy children aged 6 months–17 years [16]. In addition, it can help children overcome the fear of pain and needles [17] and save administration time [18]. However, there were also concerns over administration cost, logistical burden, and constraints on medical eligibility of the children when LAIV was provided [19,20]. While most SIVP in the United States provided LAIV as the sole choice or together with IIV [6,21,22], the SIVP in Hong Kong mainly provided IIV [23]. Second, vaccination was mainly provided by school nurses of clinics set at schools in the United States [24,25], while in Hong Kong, vaccination was provided by a vaccination outreach team through public-private-partnership or the Department of Health (DH) [23]. Third, in Hong Kong, only around 17% of the special and international elementary schools received services from school nurses [26], while most elementary schools did not have a school nurse to support the SIVP [27]. In the United States, the school nurses were responsible for checking the consent forms and screening for students’ health eligibility to vaccination [22], while in Hong Kong, this may become the responsibility of school staff without the support of a school nurse, which may be perceived as a burden to school staff. Furthermore, non-return of parental consent forms was identified to be an important challenge of implementing SIVP in the United States, which was linked to context-based communication and the consent process [8,9,22], but how SIVP was communicated with parents and students via schools and the consent process in the context of an Asian city remained unexplored. Overall, the contexts of mainly providing IIV for school-aged children and having school staff instead of school nurses for the coordination of the school-located immunization programs in Hong Kong were similar to those in other Asian countries such as Japan and Korea [28,29,30]. Therefore, learning elementary schools’ experience with SIVP in Hong Kong may help to generalize the findings to other Asian cities with similar contexts.

### 1.2. Study Objectives

This study was a mixed-methods study involving both quantitative and qualitative research methods, aiming to examine the acceptability, delivery challenges, and communication about SIVP from the perspectives of elementary school staff after the program had been implemented in Hong Kong for two years. Specifically, the study was aimed to answer the following research questions:

1. What were the attitudes about and intention to initiate or continue the implementation of SIVP among elementary school staff?
2. What were the delivery challenges experienced by school staff over the past two years of program implementation?
3. How were SIVP communicated with parents and students via schools, and how was the consent process implemented?

For the quantitative part (questionnaire-based survey), we hypothesized that schools that had not implemented SIVP would have lower positive attitudes about SIVP, perceive greater difficulty in implementing SIVP, and have lower intention to implement SIVP in subsequent years compared with schools that had implemented SIVP. For the qualitative part (analysis of the in-depth interviews and school newsletter contents), since the research questions were exploratory in nature, no hypotheses were set.

## 2. Materials and Methods

This study comprised three parts: Part I. A questionnaire-based survey to evaluate elementary school staff’s acceptability (willingness to implement SIVP and their attitudes toward SIVP) and perceived challenges for the implementation of SIVP; Part II. A qualitative study involving in-depth interviews with school staff who had been involved in SIVP to explore their acceptability, perceived delivery challenges, and communication about SIVP, and Part III. Content analysis of publicly accessible school newsletters to inform communication about SIVP with parents via schools and the consent process. School staff’s preference for LAIV versus IIV was also examined in both Part I and Part II.

### 2.1. Participants and Data Collection

#### 2.1.1. Questionnaire-Based Survey

Quantitative data were collected from December 2019 to May 2020 using an online questionnaire to assess elementary school staff’s attitudes toward childhood SIV and SIVP, intention to initiate or continue the implementation of SIVP, perceived challenges for the delivery of SIVP (Details of the items were available in Supplementary Table S1), and preference for LAIV and IIV. Furthermore, demographic data were collected. All staff of elementary schools in Hong Kong who could read the online Chinese or English questionnaire and provided consent to participate in the survey were eligible subjects. Subjects were first recruited by sending an invitation letter briefing the purposes of the survey attached with a hyperlink of an electronic questionnaire to the school principals via the emails identified from the school websites. The contact person of each school was encouraged to distribute the questionnaire to other school staff, including the school nurse, if applicable. A follow-up call was made for schools that didn’t respond to the survey three days after the invitation letter was sent. Since all elementary schools were closed due to the COVID-19 pandemic during the data collection period, this sampling method encountered difficulty in reaching sufficient subjects. Therefore, the questionnaire was also delivered via the Hong Kong Professional Teachers’ Union to reach more potential subjects. All participants indicated their consent to participate in the survey after reading the information sheet on the cover page of the online questionnaire before they proceeded to the survey part. A food coupon valued at USD 6.40 was provided for each participant who completed the questionnaire to improve the response rate.

#### 2.1.2. In-Depth Interviews

School staff who had various roles in the implementation of SIVP (e.g., decision-making for program implementation, coordination, communication, and assisting and supporting) were recruited to participate in the one-to-one in-depth interview via telephone. Participants were identified from the survey part, which indicated that they were involved in SIVP and were willing to participate in the in-depth interview. Purposive sampling was used to maximize heterogeneity in participants’ backgrounds (e.g., type of school, type of influenza vaccine provided in SIVP, role in SIVP, age, and sex). During the interview, participants were asked about their experiences in implementing, coordinating, communicating about, and supporting the SIVP. Oral consent was obtained from each participant before the interview formally started. All interviews were conducted in April–June 2020. Each interview lasted for ~20 min and was audio taped. Data collection stopped when we achieved data saturation, which was defined as no new themes emerging in the last three consecutive interviews. A supermarket coupon valued at USD $13 was provided for each participant to compensate for their time.

#### 2.1.3. Retrieving School Newsletters

School newsletters were commonly used to notify parents about SIVP and delivered to parents through an e-Class Parent App, a platform widely used by schools to communicate with parents in Hong Kong. While the e-Class platform is not publicly accessible, most schools also posted these newsletters to their school websites that were publicly accessible. Therefore, school newsletters about SIVP were retrieved from school websites for content analysis as a supplementary part to understand how SIVP was communicated with parents and the process of obtaining parental consent.

### 2.2. Data Analysis

For the survey data, Pearson chi-square test (for categorical variables) or *t*-test (for continuous variables) was conducted to compare school staff’s attitudes toward childhood SIV and perceived delivery challenges of SIVP by whether SIVP was implemented in their schools in 2019–2020. We also described participants’ perceptions of LAIV vs. IIV based on the survey data. The quantitative data analyses were conducted using SPSS 26.0 (IBM, Armonk, NY, USA). All in-depth interviews were transcribed verbatim and checked for accuracy in transcription before analysis. The transcripts were analyzed using thematic coding. Two researchers independently worked on the interview data to generate codes based on repeatedly reading the transcripts. News codes were allowed to be freely generated based on the interpretation of the data. Each researcher compared the newly generated codes and old codes constantly to ensure that all codes generated were mutually exclusive. Then, all codes were organized to develop theoretical categories. The connections among categories were subsequently examined to develop research themes. A third researcher was involved in checking the interpretation of the data and the quotations for illustrating the research themes and categories to ensure consistent interpretation of the data from different perspectives. For the content analysis of the school newsletters, a tentative coding scheme was first drafted based on analysis of a random subset of 10% of the retrieved newsletters. Then, two researchers independently coded all retrieved newsletters. Codes were finally sorted and organized to develop thematic categories to describe the major pattern of data. Interrater reliability was evaluated by calculating Cohen Kappa with a value of ≥0.6, indicating moderate reliability [31]. Disagreements in coding were solved by a joint discussion of the two coders with the third researcher and going back to the original data. Analyses of the in-depth interviews and the school newsletters and checking the interrater reliability was conducted using NVivo 12.0 (QSR International, Melbourne, Australia).

## 3. Results

### 3.1. Questionnaire-Based Survey

A total of 405 questionnaires were obtained, of which 25 were excluded because over 50% of data in the questionnaire were missing, leaving 380 for data analysis. Since the survey was anonymous, we were not able to distinguish participants by their affiliated schools. However, we inferred that school staff of at least 40 elementary schools was covered in the survey based on the e-mail addresses provided by participants (for the delivery of incentives) and the number of school principals being covered. Most participants were female and teachers (Table 1). Of the 380 participants, 36.3% (138/380) reported that their schools implemented SIVP in both 2018–2019 and 2019–2020, 42.9% (163/380) started to implement SIVP in 2019–2020, and 5.8% (22/380) implement the program in 2018–2019 but discontinued in 2019–2020. Most believed that their schools would be likely/very likely/certain to implement SIVP in the next year (92.4%, 351/380), and attitudes toward SIVP were overall positive with a mean score of 4.05 (SD = 0.67) for a score range of 1–5 (details of attitude item scores were presented in Supplementary Table S1). Participants whose schools implemented SIVP in 2019–2020 had greater intention to implement SIVP in the next year compared with participants whose schools had never implemented SIVP or implemented in 2018–2019 but discontinued in 2019–2020, but their attitudes toward SIVP did not differ by whether their schools had implemented the 2019–2020 SIVP (Supplementary Table S2). However, participants whose schools did not implement the 2019–2020 SIVP overall perceived more difficulty in the logistical arrangement for SIVP. Specifically, participants whose school did not implement the 2019–2020 SIVP perceived significantly greater difficulty in screening for students’ health eligibility for vaccination, handling students’ immediate reactions (e.g., pain, side effects) and absenteeism from classes after vaccination, communicating with parents about SIVP and arranging a suitable location for students’ vaccination (Supplementary Table S2).

**Figure 1:**
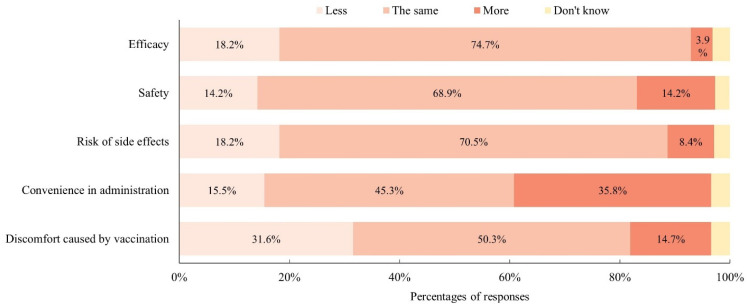
Perceptions of LAIV versus IIV among the participants (*N* = 380).

Of those whose schools had implemented SIVP, 88.2% (285/323) indicated that their schools provided IIV while 5.3% (17/323) indicated that their schools provided LAIV, with the remaining providing no data about the type of influenza vaccine used in their SIVP. All 380 participants were asked about their perceptions of LAIV versus IIV. Most participants did not differentiate their perceptions of LAIV from IIV in terms of efficacy, safety, and risk of side effects, but more than 30% of the participants believed that LAIV was more convenient and caused less discomfort in children when administered (Figure 1). The major concern for LAIV versus IIV was its efficacy (18.2%).

### 3.2. In-Depth Interviews

A total of 23 in-depth interviews were completed. Overall, three school principals, six administrators, and 14 teachers were interviewed. Three participants were from special elementary schools that provided services and education for children with intellectual disabilities, one participant was from a private elementary school, and the remaining were from government-subsidized schools (Table 2).

#### 3.2.1. Acceptability of SIVP

All participants had an overall positive attitude about SIVP. Participants generally believed that SIVP was beneficial to students and school. These beliefs were strengthened by witnessing a reduction in influenza-like illness in students and school staff after introducing SIVP.

E.g., “It was obvious that the number of sickness in teachers or students due to influenza dropped dramatically (this year) compared with that in 2017–2018 after the programme was launched.” (SD7).

Most participants believed that SIVP provided convenience for parents and hence was highly acceptable for parents.

E.g., “I feel like the most common reasons for why parents didn’t want to get their children’s vaccination from private clinics is firstly, because of money and the second reason is time. To be honest, the kids most of the time can only go to the clinics on weekends when doctors are very busy. Thus, if the vaccine is provided at schools, parents may find it a more promising way because they don’t have to spend extra time for that, and teachers can take care of the students” (SD2).

Most participants accepted SIVP to be routinized to ensure annual protection gained by children. Some believed that SIVP should be extended to cover more eligible people such as siblings of the students, parents, and school staff.

E.g., “Actually, even after children have been vaccinated, the protection cannot achieve 100%. Then will it be better if the programme can cover all personnel at schools?” (SD17).

However, participants were cautious about making SVIP compulsory for children and believed that it was important to respect individual parents’ and teachers’ concerns over the vaccines, e.g., “I think no matter taking vaccine or medicine, all consist of medical components, so I think it shouldn’t be mandatory to get the vaccine”.

#### 3.2.2. Challenges in Program Delivery

Logistical arrangement: Participants all indicated high confidence in the logistical arrangement of SIVP due to their prior experience with other school-based vaccination programs. However, they did perceive some challenges in logistics. First, most raised concerns over the burden of paperwork, particularly in the first year of implementing SIVP. The paperwork involved collecting and checking parental consent forms and other documents such as students’ vaccination cards and birth certificates to verify students’ eligibility for vaccination. Some participants mentioned that the consent form was complicated, and they did not have the medical knowledge to check the consent form and screening for students’ eligibility to vaccination.

E.g., “The (consent) form should be simple, but they made it complicated. Then the parents also felt very annoyed. Then this became a trouble for us. First, parents would call us when they didn’t know how to fill the form and second, the forms we collected were very messy. Actually, we also don’t know how to fill the form.” (SD4).

Some participants believed that obtaining parental consent and checking other documents for vaccination eligibility were not the responsibility of schools and preferred to shift the responsibility to vaccine service providers.

E.g., “I should say, it was a bit unhappy in the first year (when this program was run). We feel that it gives school too much responsibility (for paperwork), because we feel that it is them (the clinics) to earn the money, and there should be no reason for the school to take the responsibility for the clinics.” (SD7).

The experience became more positive in the second year after passing the paperwork to the vaccine service providers and the DH simplified the procedure.

E.g., “In 2019, the DH decided that the administrative work (paperwork) was passed to the medical institution to reduce our burden. Then I feel it is simpler.” (SD11).

Second, collaboration with vaccine service providers was described as a running-in process by participants whose schools had implemented SIVP for two years. Difficulties in collaborating with the vaccine service providers mainly included lacking clear guidelines about responsibility, inaccessibility to communicating with the vaccine service providers about vaccination arrangement and insufficient manpower to administer the vaccination to children, which prolonged the time occupied by vaccination.

Third, some participants also mentioned the challenges for arranging a suitable time for students’ vaccination. Arranging vaccination time required consideration of the availability of vaccine service providers, major school events (e.g., examination and other vaccination programs), the availability of vaccination venues, and the required time to obtain parental consent and for children to generate antibody after vaccination before influenza season. Balancing these factors was perceived to be challenging.

E.g., “We yet to arrange Diphtheria-Tetanus-Pertussis vaccine and measles vaccine that are required by Center for Health Protection for the students while class was suspended due to flu outbreaks. Thus, students need to get these vaccines in a very short time later… I have no idea how the actual operations would be.” (SD5).

Schools that had limited space perceived greater difficulty in determining a suitable date for students’ vaccination because the vaccination venue may be occupied by multiple events.

E.g., “We arranged the vaccination site in a music classroom because our school doesn’t have a hall. That means we need to empty one place for students to take vaccination, but all our classrooms are occupied…” (SD9).

Early planning was mentioned to be crucial.

E.g., “Maybe before the influenza season comes, we hope that the vaccine can take effect to protect the children. We always think that the earlier the arrangement is the better it is.” (SD23).

Managing students’ vaccination-related anxiety and reactions after vaccination: Most participants indicated no challenge for managing students’ anxiety before vaccination. However, participants of special schools indicated more difficulty in managing students’ vaccination-related anxiety because students with intellectual disabilities had more difficulties in making sense of the situation and understanding the information.

E.g., “for children who are moderate mentally retarded, their comprehensibility is relatively poor if you explain (the vaccination) to them. They don’t know how scared they are.” (SD10).

Some students may refuse vaccination due to extreme anxiety even parental consent had been obtained.

E.g., “I estimate that around 20–30% of the students would be more difficult to be persuaded for taking the vaccine, particular those who had autism” (SD18).

All participants perceived minimal disruption of vaccine side effects to school and classes.

E.g., “There were not so many students feeling unwell after vaccination but normally one or two would feel unwell. The reactions were very regular and normal” (SD02).

Some strategies were used to minimize the impact of vaccine side effects, such as arranging Friday to be the vaccination day, taking short observations immediately after vaccination, and arranging for younger students to be vaccinated first to allow sufficient time to observe younger students’ reactions.

E.g., “we usually arranged the lower-grade students to take the vaccination first so that the school has more time to handle their reactions if they really have the reactions (after vaccination)” (SD16).

Arranging SIVP as teamwork: The experience with SIVP was described as teamwork because it involved negotiations among school staff, vaccine service providers, and parents. The negotiation process was mainly aimed to prepare a suitable time and place for students’ vaccination accommodating multiple stakeholders’ needs such as parental consent, vaccine service providers’ availability, and school schedules. Multiple stakeholders’ engagement was believed to be essential. Commonly, it required one school staff as the coordinator, the school principal to make the final decision for administrative arrangements within the school, other teachers to distribute, collect and check parental consent forms and other documents, take students to the vaccination site and manage students’ vaccination-related anxiety, and non-teaching supporting staff and parent volunteers to set and clean the vaccination site and manage students’ vaccination order and anxiety.

#### 3.2.3. Communication with Parents and Students about SIVP

Communicating with parents to confirm their decision for children’s vaccination was a central task of the school. However, most participants seemed to be passive regarding communication with parents about the influenza vaccine. Some participants felt that it was not their responsibility to answer parents’ questions about the vaccine, and they tend to direct parents’ questions to the vaccine service providers.

E.g., “Usually parents will ask me, like whether his/her child is eligible for vaccination if child has food allergy or if the child is taking other medicine. I cannot answer such questions as it requires professional knowledge. So, I will tell them to call the medical institution for their suggestions.” (SD14).

Most participants believed that the consent form had provided sufficient information about the vaccine and hence no need to provide more explanations. Some participants felt that the school should avoid active promotion of vaccination to avoid pressuring parents’ choice.

E.g., “The school didn’t explain (why it is better to take two doses of the vaccines for the first time). We will respect parents’ choice, so we have no special suggestions for whether parents should do or not to do something” (SD13).

Most participants appeared inactive in communicating with students about the influenza vaccine. They generally believed that students were not the main decision makers for vaccination and had difficulties in understanding the information.

E.g., “No need to communicate with children because they cannot understand these things. Also, they cannot make the final decision, it is just a waste of time…” (SD22).

However, two participants mentioned the importance of more communication with younger students or students with intellectual difficulties before vaccination to relieve their anxiety.

#### 3.2.4. Preference for Type of Influenza Vaccine

Most participants indicated preferring IIV because it was believed to be more traditional, more effective, free, and more accessible (i.e., fewer constraints for eligibility) and more convenient in administration. One participant mentioned that parents were more familiar with the injectable vaccine, and hence, it was easier for teachers to explain the vaccine to parents.

E.g., “We choose it (injectable vaccine) because the children have got used to injection since they were very young…it appears that we don’t need to explain much (to the parents) before parents can understand what it is.” (SD22).

Participants also mentioned their concerns over LAIV, including its efficacy, side effect, inaccessibility to some students due to medical conditions, higher cost, and causing more discomfort in children in administration.

E.g., “For me, I think that the injectable vaccine is 100% effective, but I don’t think the intranasal vaccine will be sufficiently effective.” (SD05).

Three participants whose schools provided LAIV in the SIVP indicated a preference for LAIV and believed that LIAV induced less pain in children, was more convenient in administration and less invasive, and had fewer side effects.

E.g., “I feel that the intranasal vaccine is not so invasive and it appears that it is more acceptable for both children and parents. And I also feel that it is much quick in administration.” (SD23).

### 3.3. Content Analysis of School Newsletters about SIVP

A total of 105 school newsletters about SIVP from 539 schools that joined the 2019–2020 SIVP were retrieved, each from one school. Major categories identified from the content analysis of the retrieved newsletters are shown in Table 3. Of the 105 schools, 90 (85.7%) delivered the school newsletters with the DH’s consent form in the attachment, of which around one-half (47/90, 52.2%) required parents to return the signed consent forms regardless of accepting or rejecting the vaccine (Type A) while another half (43/90, 47.8%) only required parents who accepted the vaccine to return the signed consent forms (Type B). Around 14.2% (15/105) solely delivered the school newsletters about SIVP to parents but mentioned that the DH’s consent form would be delivered to parents who returned the reply slip of the school newsletter to indicate their consent to accept the vaccine (Type C). Details of the consent form delivery can be found in Figure S1. All the 105 school newsletters provided information about the logistical arrangement of SIVP; around 70% mentioned encouraging children to take SIV; slightly less than one-half provided information about the benefits of taking SIV, and around 20% about the risk of influenza to children. However, only several schools provided information about vaccine safety, and only one provided information about the effectiveness of SIVP after running SIVP for the first year (Table 3). Examples of quotes for major thematic categories are shown in Supplementary Table S3.

## 4. Discussion

Elementary school staff in Hong Kong overall had positive attitudes toward SIVP. Most participants in the survey believed that SIVP was beneficial to students and school and highly acceptable for parents. Witnessing reduction in influenza-like illnesses and high parental acceptability after implementing SIVP seemed to strengthen such positive attitudes. While current SIVP mainly focused on providing SIV for elementary students, the in-depth interviews suggest that SIVP may be expanded to cover school staff, parents, and students’ siblings who are eligible for receiving government subsidies for SIV to maximize its coverage. Despite high acceptability for the routinization of SIVP, school staff preferred to make SIV optional rather than compulsory for students to respect individual parents’ and teachers’ concerns over SIV.

Schools with lower intention to implement SIVP were comparable to those with higher intention to implement SIVP in terms of their attitudes toward SIVP but perceived more logistical difficulties in the delivery of the program. While positive experience with previous school-located vaccination programs can enhance confidence in logistical arrangements for SIVP, schools’ limited infrastructure could hinder the implementation of SIVP [32]. For instance, schools that have limited space for setting the vaccination site are less flexible for time arrangement and hence more difficult to determine a suitable vaccination date because determining a suitable vaccination date requires accommodation of competing stakeholders’ needs for arranging other school events. Moreover, planning and coordinating SIVP was a school teamwork that involved multiple school staff including administrative, teaching, and non-teaching staff. Schools that were insufficiently staffed may encounter more difficulties in coordinating the program [32]. Practical support such as a mobile vaccination station and a collaborative group of volunteers [33] should be provided for schools that have limited space and are insufficient staffing.

Despite overall high confidence in the logistical arrangement for SIVP, elementary schools that had implemented SIVP still encountered major difficulties that were locally relevant. First, while other studies reported the challenges of low returned rates of signed parental consent forms [8,9,34], this was not a concern in Hong Kong. Instead, our study found that school staff were mainly concerned about their capacity to check parental consent forms and documents to support students’ eligibility to vaccination and answer parents’ inquiries about vaccines. Some questioned that these tasks may be out of their responsibility scope. Clarification of the responsibility between vaccine service providers and schools and minimizing schools’ burden of paperwork are essential to improve schools’ experience with SIVP and their satisfaction with vaccine service providers, and thereby promote the program sustainability. Second, fear of being overwhelmed by the requirement to complete multiple vaccination programs within a short period was identified among school staff. This could become particularly obvious when schedules of the vaccination programs were disrupted by the COVID-19 pandemic [35] or other events. A good partnership among government, vaccine service providers, and schools should be established to facilitate the early planning of multiple school-located vaccination programs [36]. Third, schools that provide services for students with intellectual disabilities perceived more difficulties in managing students’ vaccination-related anxiety due to students’ intellectual difficulties in understanding the vaccines and the situation. Completion of vaccination could fail in extremely anxious students even parental consent for vaccination had been obtained. This could be a concern because conditions of intellectual disabilities are associated with a high risk for complications due to influenza virus infection [37]. Managing special students’ anxiety may require extensive volunteer support, greater effort in communicating with children prior to vaccination, and introducing an intranasal vaccine to reduce fear of needles [8,38].

Our study revealed that school staff were generally passive in communicating with parents about influenza vaccines. This may be attributed to their perceived inadequate medical knowledge and feeling of discomfort to share their attitudes toward influenza vaccines, which were believed to pressure parents’ vaccination decisions. Less than 10% of the sampled school newsletters mentioned the safety of influenza vaccines, indicating that schools may be unconfident about the safety of influenza or avoid putting themselves in a dilemma once rare serious vaccine adverse effects occur. Although the informed consent form was believed to be an important source of information about influenza vaccines for parents, not all schools required parents to return the consent forms or distribute the consent forms to parents for their vaccination decision. These consent processes cannot ensure parents read or even have access to the information stated in the consent form before they make the decision for their children’s vaccination, harming the informed decision-making for childhood SIV [39]. Parents who could not obtain supportive information from the consent form may seek information from the Internet to guide their decision and thus could be problematic as misinformation is widespread on the Internet [40]. Furthermore, the need to communicate with students about influenza vaccines was generally overlooked by school personnel. While young students are not the final decision makers for their own SIV, students’ insufficient understanding of the benefit and safety of influenza vaccine may increase their anxiety about vaccination and their failure to complete the vaccination [34].

Finally, the survey data found that LAIV was perceived to be comparable to IIV in terms of vaccine efficacy, side effects, and safety but be more convenient and cause less discomfort in administration. However, the qualitative data revealed that schools that used IIV in their SIVP may avoid changing the type of vaccine to avoid confusing parents and the burden of giving additional explanations. IIV was perceived to be more traditional, familiar to students, and more accessible to students due to fewer constraints in medical conditions. There seemed to be a misperception about the efficacy of LAIV due to its unconventional administrative method. However, schools that had provided LAIV in their SIVP were more positive about LAIV. In the United States, LAIV was perceived to cause less anxiety in students and save more time in administration [8,41]. These advantages make LAIV a promising vaccine for students who have needle phobia and schools that have limited infrastructure to implement SIVP. LAIV may be introduced as one option for SIVP to increase schools’ acceptability.

## 5. Limitations

These studies had several limitations. First, despite the greater effort made to reach all potential subjects in Hong Kong for the questionnaire survey, the survey was eventually conducted using convenience sampling due to the disruption of the COVID-19 pandemic and thereby could not estimate the response rate and evaluate the non-response bias. Second, our study required the participants to recall their experience with SIVP, and hence, there could be recall biases in the survey and in-depth interview data. Third, only school newsletters that were publicly available were retrieved for data analysis, which limited the analysis to merely 20% of the schools that had participated in SIVP. Fourth, only one school staff from private schools was recruited for the qualitative interview. The perspectives of private school staff on SIVP may not have been fully explored. In addition, due to the COVID-19 pandemic, all in-depth interviews were conducted via telephone, which hindered observations of participants’ non-verbal languages during the interviews. Fifth, this study mainly evaluated the implementation of SIVP from the perspectives of the school staff. Future studies should explore the perspectives of other stakeholders such as vaccine service providers and parents (or school staff with children) regarding the implementation of SIVP. Furthermore, the generalizability of the study findings may be limited by context, but the findings should provide valuable insights into the implementation and delivery of SIVP in cities with similar contexts.

## 6. Conclusions

Clarification of responsibility between vaccine service providers and schools and minimizing school staff’s workload in obtaining parental consent and checking students’ vaccination eligibility may be essential to improve school staff’s experience with SIVP and satisfaction with the collaboration with vaccine service providers. Extensive support such as volunteer supports, mobile vaccination stations, and providing the intranasal vaccines as an option may be needed to support schools with limited infrastructure for the implementation and delivery of SIVP and the vaccination for students with intellectual disabilities. Training may be provided to school staff involved in SIVP to enhance their awareness, skills, and confidence in communicating with parents and students about the influenza vaccine. Finally, the parental consent process should be standardized to ensure parents can access information in the informed consent forms before making the decision for their children’s vaccination.

## Figures and Tables

**Table 1:** Demographics of participants in the questionnaire survey (*N* = 380).

Characteristics	*N* (%)
Sex	
Female	299 (78.7)
Male	70 (18.4)
Missing	11 (2.9)
Age group (years)	
18–24	9 (2.4)
25–34	123 (32.4)
35–44	125 (32.9)
45–54	86 (22.6)
55 or above	25 (6.6)
Missing	12 (3.2)
Marital status	
Single	151 (39.7)
Married	218 (57.4)
Divorced/Separated/Widowed	5 (1.4)
Missing	6 (1.6)
Educational attainment	
Primary or below	2 (0.5)
Secondary or matriculation	13 (3.4)
Tertiary	362 (95.3)
Missing	3 (0.8)
Types of participants’ school	
Full-government-subsidized school	330 (86.8)
Partial-government-subsidized school	20 (5.3)
Private school	29 (7.6)
Missing	1 (0.3)
Role of participants at school	
School principal	18 (4.7)
Administrator	70 (18.4)
Teacher	275 (72.4)
School nurse/social worker/teacher assistant	15 (4.0)
Missing	2 (0.5)
Year of working experience (years)	
0–5	151 (39.7)
5–10	65 (17.1)
10–20	92 (24.2)
≥20	67 (17.6)
Missing	5 (1.3)
Monthly income (HKD) ^a^	
≤19,999	36 (9.5)
20,000–39,999	105 (27.6)
40,000 or above	231 (60.8)
Missing/Refused	8 (2.1)

^a^ 1 HKD = 0.13 USD.

**Table 2:** Demographics of school personnel who participated in the in-depth interviews.

ID	Sex	Age Groups	2018–2019	2019–2020	School Type	Position	Working Years
SD1	F	45–54	NA	IIV	AS	Admin	>20
SD2	F	45–54	L	IIV	AS	Teacher	10–20
SD3	M	45–54	L	LAIV	AS	Admin	>20
SD4	F	≥55	I	IIV	AS	Admin	>20
SD5	F	35–44	I	IIV	AS	Teacher	10–20
SD6	F	45–54	I	IIV	AS	Principal	0–5
SD7	F	45–54	I	IIV	PS	Principle	>20
SD8	F	25–34	L	LAIV	AS	Teacher	0–5
SD9	F	45–54	L	LAIV	AS	Teacher	>20
SD10	F	45–54	I	IIV	SS	Teacher	>20
SD11	F	35–44	I	IIV	AS	Teacher	10–20
SD12	F	25–34	I	IIV	AS	Teacher	0–5
SD13	M	35–44	I	IIV	AS	Teacher	5–10
SD14	F	35–44	I	IIV	SS	Teacher	0–5
SD15	F	55 or above	I	IIV	AS	Principle	10–20
SD16	F	35–44	NA	IIV	AS	Admin	>20
SD17	F	35–44	I	IIV	AS	Teacher	10–20
SD18	F	45–54	I	IIV	SS	Teacher	5–10
SD19	F	45–54	NA	IIV	AS	Teacher	>20
SD20	M	35–44	I	IIV	AS	Admin	10–20
SD21	F	25–34	L	LAIV	AS	Teacher	10–20
SD22	F	25–34	I	IIV	AS	Teacher	0–5
SD23	M	45–54	NA	LAIV	AS	Admin	10–20

ID refers to subject identification, which is presented in the format of SDXX. M: male; F: female; IIV: inactivated influenza vaccine; LAIV: live, attenuated influenza vaccine; NA: not provided influenza vaccine; AS: aided school; PS: private school; SS: special school; admin: administrator.

**Table 3:** Thematic category and their frequency identified from the retrieved school newsletters (*N* = 105).

	Number of Schools (%)
The process of obtaining parents’ consent	
Type A	47 (44.8)
Type B	43 (41.0)
Type C	15 (14.2)
Mentioning logistical arrangement of SIVP (e.g., time, procedure)	105 (100.0)
Indicating positive attitudes about childhood SIV (e.g., “encourage”)	72 (68.6)
Mentioning benefit of taking SIV	49 (46.7)
Individual benefit (e.g., reduce sickness and absenteeism)	28 (26.7)
Social benefit (community benefit)	21 (20.0)
Mentioning risk of influenza to children	24 (22.9)
The severe consequences of influenza virus infection	14 (13.3)
Children’s high susceptibility to influenza infection at school	21 (20.0)
Mentioning vaccine contraindication and eligibility for taking SIV	20 (19.0)
Mentioning that SIV was recommended by health professional	15 (14.3)
Mentioning that influenza vaccine is safe	7 (6.7)
Mentioning the positive effect after introducing SIVP (e.g., reduction in students’ sickness due to influenza)	1 (1.0)

## Data Availability

The data are available under reasonable request to the corresponding authors.

## Supplementary Materials

The following are available online at https://www.mdpi.com/article/10.3390/vaccines9101175/s1, Figure S1: Type of schools based on their procedure to obtain parental consent for children’s influenza vaccination, Table S1: Items for measuring attitudes, intention, and perceived challenges regarding the implementation of SIVP, Table S2: Comparison of participants’ intention to participate in SIVP, attitudes, and experience by whether SIVP was implemented in 2019–2020 in participants’ schools, Table S3: Thematic category and examples of quotes from school newsletters.
