# Supplementary material for: A Mixed-Methods Study to Evaluate Elementary School Staff’s Acceptability, Delivery Challenges, and Communication Regarding the Implementation of School-Located Influenza Vaccination Program in Hong Kong
Source: Vaccines (Basel). 2021 Oct 14;9(10):1175. doi: 10.3390/vaccines9101175 (PMC8540161; doi:10.3390/vaccines9101175)
Supplement: Supplementary file 1 [file vaccines-09-01175-s001.zip › vaccines-1298278-supplementary.pdf]

vaccines

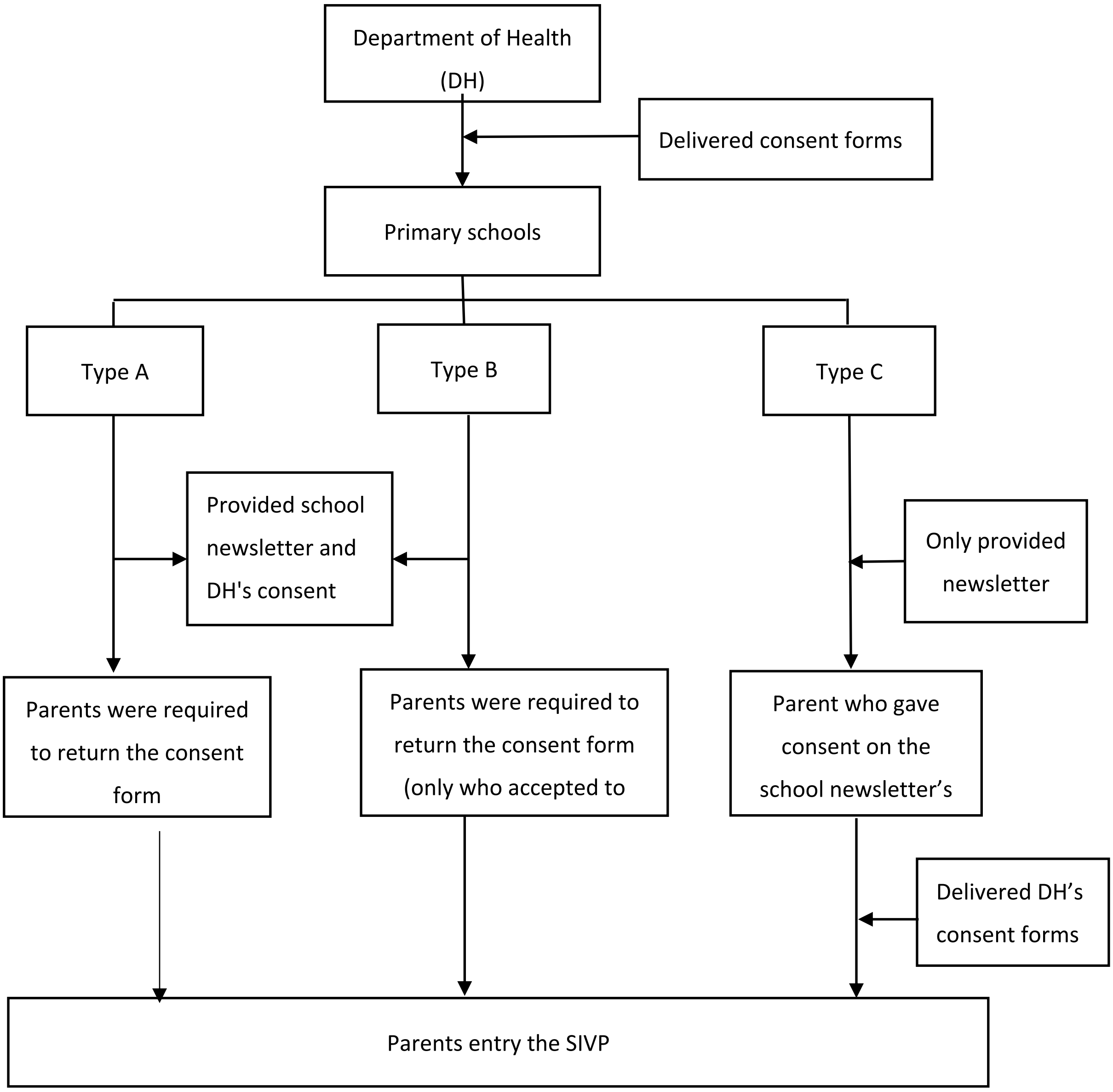

Figure S1. Type of schools based on their procedure to obtain parental consent for children's influenza vaccination. Type A example: "After carefully reading all the information, parents should fill in the return receipt (in the newsletter) and the injection forms (consent or refusal form), no matter agree or disagree to join the SIVP programme". Type B example: "After carefully reading all the information, parents should fill in the return receipt (in the newsletter) and the consent form, if you are interested to join the SIVP programme". Type C example: "If you are interested in joining in the SIVP, please fill in and return the receipt. After school collecting the receipts, a consent form will be sent to parents who are willing to join the programme".

Table S1. Items for measuring attitudes, intention, and perceived challenges regarding the implementation of SIVP.

**Statements**	**Response scale**	**Mean score (SD)**
Attitudes items		
Influenza vaccination is important for primary school students	1–5 agreement scale	4.22 (0.80)
Influenza vaccination can reduce students' risk of getting flu	1–5 agreement scale	4.20 (0.79)
Introducing SIVP is a good idea for reducing the chance of having a flu outbreak in school	1–5 agreement scale	4.20 (0.79)
Influenza vaccination can reduce students' risk of developing complications even if he/she got flu	1–5 agreement scale	4.06 (0.81)
Influenza vaccination can reduce school staff's risk of getting flu	1–5 agreement scale	4.03 (0.86)
The SIVP is effective for reducing students' absenteeism due to influenza	1–5 agreement scale	3.94 (0.86)
The SIVP is effective for reducing school staff's absenteeism due to influenza	1–5 agreement scale	3.78 (0.91)
Overall, I feel that SIVP is beneficial to students, school and the whole society	1–5 agreement scale	4.15 (0.75)
Overall, I think the influenza vaccine is safe for my students	1–5 agreement scale	4.06 (0.75)
Overall, I believe that most parents will accept SIVP	1–5 agreement scale	3.98 (0.70)
Perceived challenges items		
Screening for students' medical eligibility to vaccination	1–4 difficulty scale	2.35 (0.74)
Arranging time for vaccine administration	1–4 difficulty scale	2.24 (0.73)
Arranging personnel to coordinate SIVP	1–4 difficulty scale	2.22 (0.72)
Coordinating parents to be on site at the vaccination day	1–4 difficulty scale	2.21 (0.75)
Relieving students' anxiety immediately before vaccination	1–4 difficulty scale	2.20 (0.63)
Handling students' reactions (e.g., pain, side effects) immediately after vaccination	1–4 difficulty scale	2.20 (0.63)
Training school staff on coordinating SIVP	1–4 difficulty scale	2.13 (0.69)
Obtaining parent consent forms	1–4 difficulty scale	2.11 (0.68)
Handling students' absenteeism after the vaccination	1–4 difficulty scale	2.07 (0.64)
Communicating with parents about SIVP	1–4 difficulty scale	2.03 (0.63)
Arranging a suitable location for students' vaccination	1–4 difficulty scale	1.85 (0.69)
Overall evaluation of the whole process	1–4 difficulty scale	2.12 (0.55)
Intention		
How likely do you think your school will participate in the SIVP next year	1–7 likelihood scale	4.72 (1.06)

Table S2. Comparison of participants' intention to participate in SIVP, attitudes and experience by whether SIVP was implemented in 2019–2020 in participants' schools (*N* =380).

Variables	Implemented in SIVP in 2019–2020 (*N* = 301)	Did not implement SIVP in 2019/2020 (*N* = 79)	Differences (p value)
Intending to implement SIVP in the next year (Likely/very likely/certain vs. Never/very unlikely/unlikely/evens).	97.3%	73.4%	<0.001 [a]
Score of Attitudes towards SIVP (Mean, SD)	4.07 (0.671)	4.00 (0.673)	0.471 [b]
I have enough information about influenza vaccine to answer parents or student's questions (Agree/strongly agree vs. strongly disagree/disagree/neutral)	43.5%	34.6%	0.155 [a]
I would like to know more information about influenza vaccine (Agree/strongly agree vs. strongly disagree/disagree/neutral)	70.8%	54.4%	0.00 6[a]
Perceived difficulties in logistical arrangement of SIVP (somewhat difficulty/very difficult vs. Not at all difficult/not very difficult)			
Screening for students' medical eligibility to vaccination	34.1%	49.1%	0.013 [a]
Arranging time for vaccine administration	31.8%	34.2%	0.695 [a]
Arranging personnel to coordinate SIVP	29.1%	39.2%	0.085 [a]
Coordinating parents to be on site at the vaccination day	29.1%	36.7%	0.194 [a]
Relieving students' anxiety immediately before vaccination	25.6%	30.4%	0.393 [a]
Training school staff on coordinating SIVP	24.3%	32.9%	0.123 [a]
Obtaining parent consent forms	21.6%	27.8%	0.239 [a]
Handling students' reactions (e.g., pain, side effects) immediately after vaccination	21.5%	35.4%	0.011 [a]
Handling students' absenteeism after the vaccination	17.2%	29.1%	0.018 [a]
Communicating with parents about SIVP	16.0%	29.1%	0.008 [a]
Arranging a suitable location for students' vaccination	11.0%	23.1%	0.005 [a]
Overall evaluation of the whole process	15.0%	37.2%	<0.001 [a]

[a] Pearson chi-square test; [b] t-test

Table S3. Thematic category and examples of quotes from school newsletters

Thematic category	Example of quotes from the newsletters
Mentioning logistical arrangement of SIVP (e.g., time, procedure)	Vaccine provider: XXX Outreach (medical institution)<br>Time: Wednesday, 18th November at 10:00a.m.<br>Place of vaccination: School hall<br>Vaccine type: Inactivated influenza vaccine (IIV)<br>Fee: Free of charges for all eligible students.
Indicating positive attitudes about childhood SIV (e.g., "encourage")	To encourage school children to receive influenza vaccination, our school has joined the 2019–2020 Seasonal Influenza Vaccination School Outreach (Free of Charge) Programme subsidized by Department of Health.
Mentioning benefit of taking SIV	
Individual benefits (e.g., reduce sickness and absenteeism)	Taking the influenza vaccine is an effective mean to protect students against influenza and reduce students' sickness and school absenteeism.
Social benefit (community benefit)	Vaccinating young students at school can reduce influenza transmission in the community.
Mentioning risk of influenza to children	
The severe consequences of influenza infection	Influenza is usually more common in periods from November to March. Influenza can cause serious illness in high-risk individuals (children, elderly) and may result in life-threatening complications such as bronchitis or pneumonia.
Children's vulnerability to influenza virus infection	Due to the higher intensity of social contacts at school, children are more vulnerable to influenza infection.
Mentioning that influenza vaccine is safe	Influenza vaccination is a safe measure to prevent influenza.
Mentioning vaccine contraindication and eligibility for taking SIV	People who have a history of severe allergic reaction to any vaccine component are not recommended to receive influenza vaccine.
Mentioning that SIV was recommended by health professional	The "Scientific Committee on Vaccine Preventable Diseases" recommends children aged 6 months to 11 years as one of the priority groups.
Mentioning benefits after introducing SIVP (e.g., reduction in students' sickness due to influenza)	Based on previous experience in participating in SIVP, school found a significant drop in influenza infections than before, which indicated the influenza vaccination could be beneficial for students.
